# First Person - Gustavo Balbinot and Clarissa Pedrini Schuch

**DOI:** 10.1242/bio.054403

**Published:** 2020-08-13

## Abstract

First Person is a series of interviews with the first authors of a selection of papers published in Biology Open, helping early-career researchers promote themselves alongside their papers. Gustavo Balbinot and Clarissa Pedrini Schuch are first authors on ‘Mechanical and energetic determinants of impaired gait following stroke: segmental work and pendular energy transduction during treadmill walking’, published in BiO.

Gustavo Balbinot is a postdoctoral fellow at KITE - Toronto Rehabilitation Institute, University Health Network, Toronto, Canada. Clarissa Pedrini Schuch is a postdoctoral fellow at the Graduate Program in Rehabilitation Sciences, Federal University of Health Sciences of Porto Alegre (UFCSPA), Brazil.


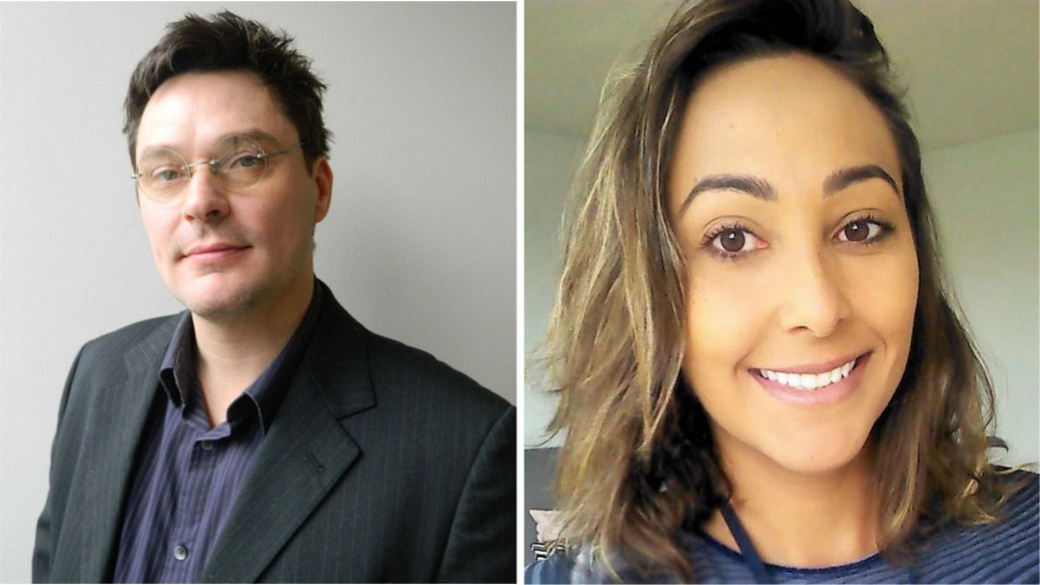


**Gustavo Balbinot and Clarissa Pedrini Schuch**

**What is your scientific background and the general focus of your lab?**

**Gustavo:** I am a postdoctoral fellow at the Rehabilitation Engineering Laboratory, which is focused on developing new technologies to assist neurorehabilitation. Specifically, my team, the Adaptive NeuroRehab Systems Lab, uses machine-learning algorithms to understand the classification of hand postures and neurophysiological data in stroke and spinal cord injury to assist neurorehabilitation. I think dealing with complex datasets and machine or deep-learning algorithms is the key to the next generation of scientific discoveries.

**Clarissa:** I am a physiotherapist trained in Brazil. I have a clinical and preclinical background in neuroscience and biomechanics. This unconventional pathway allows me to see the neurological rehabilitation process through a wide lens and provides a unique opportunity to bridge existing gaps between preclinical research and clinical practice. Specifically, my research aims at optimizing the effects of physical and cognitive rehabilitation in people with brain injuries, such as stroke and cerebral palsy. Another branch of my research is dedicated to studying the biomechanics of human walking, focusing on stroke gait mechanics and energetics, and Parkinson's disease gait.

**How would you explain the main findings of your paper to non-scientific family and friends?**

**Gustavo and Clarissa:** Some people can walk again soon after a stroke, and others may take longer to achieve this goal. People recover in different ways and to differing degrees. The outcomes of this work have helped us to understand how stroke survivors adapt their gait to maintain efficient walking. The findings of our study may assist in developing assistive devices or rehabilitation programs to reduce the metabolic burden of locomotion following stroke and increase the quality of life of stroke survivors.

“The outcomes of this work have helped us to understand how stroke survivors adapt their gait to maintain efficient walking.”

**What are the potential implications of these results for your field of research?**

**Gustavo and Clarissa:** Advances in medicine have led to many people surviving a stroke, but the prevalence of long-term disability remains high. This paper allowed us to increment the understanding of how chronic stroke survivors adapt their gait to optimize mechanical and metabolic energy conservation. The outcomes of this work may contribute to the development of better-tailored motor rehabilitation therapies to optimize walking energy conservation. In the new era of soft wearable robots and textile-based sensors, the use of ‘smart’ exosuits can potentially overcome gait deficits in stroke, ultimately reducing the metabolic cost of hemiparetic walking. The implication of our findings is in assisting engineers to fine-tune these assistive devices to reduce excessive compensation and increase the energy recovery of people living with stroke-related gait impairments.

**What has surprised you the most while conducting your research?**

**Gustavo:** The most wonderful surprise was to understand the continuum spectrum of post-stroke rehabilitation research, from animal models to humans. I was able to identify many forms to improve preclinical research focusing on the translation of findings to the final user, the stroke survivor. Every little gain is important, we are far from the cure of stroke or spinal cord injury, but little by little, it is possible to help people, to make a difference.

“…we are far from the cure of stroke or spinal cord injury, but little by little, it is possible to help people…”

**Clarissa:** I can highlight two significant aspects that fascinated me during this project. First, reading and studying the vast body of work produced by Cavagna in the 1960s and 70s regarding the pendular mechanism of human walking. After a long learning process, I was able to reproduce his methodology. It was awesome! Second, how the human locomotor system adapts to restraint imposed by brain ischemia, allowing the stroke survivors to maintain the pendular mechanism of walking.

**Figure:**
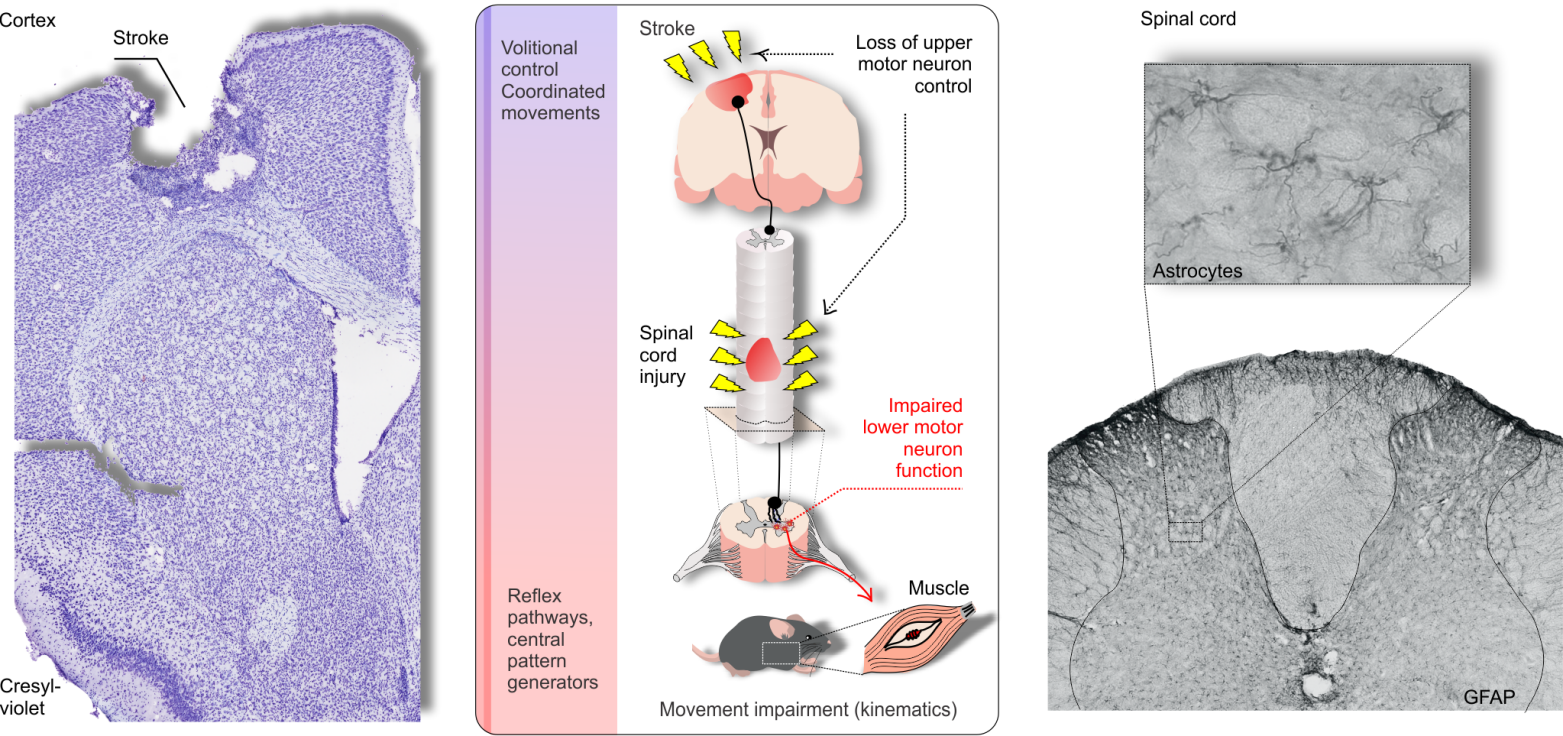
**Animal models are valuable to test the efficacy of experimental drugs or treatments.** Researchers usually induce a stroke or spinal cord lesion and test the efficacy of neurorehabilitation in increasing coordinated volitional motor control. The image on the left is from a cortical stroke induced by photothrombosis in mice, this stroke affected the upper-limb motor area (courtesy of Dr. Diane Lagace). While working with Dr. Dale Corbett and Dr. Diane Lagace at the University of Ottawa - Canada, we were pioneers in developing a kinematics-based framework to describe the motor impairments following a stroke in rodents. This work showed the first quantitative description of the “classical” flexor synergy in rodents (Balbinot et al., 2018). We believe the detailed description of movement impairment and recovery using kinematics holds the key to increase the translation of findings from animal models to the clinic. Many brain regions are activated during the recovery process to support function via true recovery of movements or compensation (Balbinot and Schuch, 2019). While compensation may increase function, true recovery of movement is the utmost goal of novel treatments to mitigate the motor impairments following a stroke or spinal cord injury. This constitutes a major gap and bias of the current animal models: the inability to distinguish between true recovery of movements or compensation, as both lead to greater functionality. While working with Dr. Matilde Achaval, Dr. Milton Antônio Zaro and Dr. Marco Aurélio Vaz at the Federal University of Porto Alegre, Brazil, we were able to assess the spinal cord pain modulation in an experimental model of knee osteoarthritis (Balbinot et al., 2019). Glial fibrillary acidic protein (GFAP) expression in spinal astrocytes is augmented during chronic pain processes, but also involved in the glial scar formation following a spinal cord injury. Interestingly, GFAP is elevated in a severity-dependent fashion in spinal cord injury and may predict injury severity (Kwon et al., 2010). The image on the right is from a previously published work (Balbinot et al., 2019).

**What, in your opinion, are some of the greatest achievements in your field and how has this influenced your research?**

**Gustavo:** I think the neurorecovery field is taking advantage of novel animal models, especially using transgenic rat and mouse models, to unveil novel drug targets or mechanisms for recovery. These models, when combined with novel ways to understand motor impairment in great detail, such as using machine learning and kinetics/kinematics approaches, may lead to important discoveries with great translational potential in the upcoming decades.

**Clarissa:** One hot topic in the stroke recovery field is the development of biomarkers for stroke recovery. The use of recovery biomarkers aligned with movement kinematic outcomes may improve the understanding of who might recover better and facilitate personalized interventions, guiding the delivery of effective treatment to the right people, at the right time.

**What changes do you think could improve the professional lives of early-career scientists?**

**Gustavo and Clarissa:** We believe that to improve the professional lives of early-career scientists, the scientific community needs more empathy. Early-careers scientists cannot survive alone, we need collaborations. Established principal investigators (PIs) usually have many students and, as such, opportunities to keep themselves productive and grow. On the other hand, the early-career scientist is one and depends solely on its own ability to finish and publish the research. We come from different parts of the world, social status and backgrounds and many times have to struggle to keep our lives and careers going. Especially for early-career scientists from developing countries, it is crucial to increase access to funding and expand the opportunities for international collaboration. In Brazil, for example, several amazing researchers are struggling to produce high-quality science due to a lack of financial resources. Profit and not-for-profit organizations could collaborate with the Universities to augment financial research support. Additionally, short-term international scholarships are an excellent way to help students to augment their academic experiences and networking.

**What's next for you?**

**Gustavo:** I will keep pursuing my postdoctoral research for the next years focusing on getting more funding and awards to enable my continuing training as a big data scientist. I wish to apply this knowledge in my future lab, which should focus on translational neuroscience to mitigate motor disorders and contribute to the testing of new experimental drugs, treatments, or assistive devices.

**Clarissa:** I will finish my postdoctoral fellowship this year and will apply for positions to continue working with stroke-related research. I will also pursue my career as a trained physiotherapist in Canada. In the following years, I wish to incorporate my research background into clinical practice in addition to continuing my research investigations in animal models and humans.
